# The effect of silver nitrate versus clotrimazole in treatment of otomycosis

**DOI:** 10.1007/s00405-026-10162-7

**Published:** 2026-04-06

**Authors:** Rami Fatouh Tantawy, Ahmed S. Elsharkawy, Hamada Hashem

**Affiliations:** 1 Otorhinolaryngology Department, Faculty of Medicine, Benha University, Benha, Qalyubia 13511 Egypt; 2 Otorhinolology Department Shebein Elkom Teatching Hospital , Menoufiya, 32512 Egypt

**Keywords:** Silver Nitrate, Clotrimazole, Otomycosis

## Abstract

**Background:**

Otomycosis is a medical condition defined as the epithelial infection of the external auditory canal caused by yeast cells and filamentous fungi, and constitutes about 9% of external otitis. The purpose of this research was to critically compare the outcomes of topical application of silver nitrate gel 0.2% versus clotrimazole cream 1% in the management of otomycosis and in decreasing the recurrence rate.

**Methods:**

This prospective comparative study included 200 adult patients with otomycosis attending the Outpatient Clinic of the Otorhinolaryngology Department at Benha University Hospitals. The cases were assigned to 2 equal groups: group (1) managed with silver nitrate 0.2% gel-soaked Gelfoam, and group (2) managed with clotrimazole 1% cream-soaked gauze. Patients were examined with an otoscope.

**Results:**

Group (1) had significantly lower edema at 3 days than group (2) (*P* = 0.018), with no significant difference between both groups at 1 day, and both had no edema at 1 week. At 3 days and 1 week, group (1) had significantly lower pain than group (2) (*P* = 0.026, 0.043), with no significant difference between the two groups at 1 day. Group (1) exhibited a significantly earlier resolving time and also a reduced recurrence rate than group (2) (*P* < 0.001, *P* < 0.001).

**Conclusions:**

In the treatment of otomycosis, both clotrimazole 1% cream and silver nitrate gel were effective. Silver nitrate gel was superior to clotrimazole, with earlier resolution and fewer/minimal recurrences.

## Introduction

Otomycosis is a medical condition characterized by an epithelial infection of the external auditory canal (EAC), caused by yeast cells and filamentous fungi, accounting for about 9% of external otitis [1]. Tinnitus, decreased hearing, blockage, ear pain, and ear discharge are among the most prevalent symptoms of otomycosis. As the most common symptoms of otomycosis, otalgia and otorrhea, are nonspecific, maintaining a high index of suspicion is critical for its precise diagnosis [2]. The presence of seborrheic dermatitis, cerumen, genetic factors, antibiotic or steroid therapy use for long duration, an impaired immune system, lack of hygiene, a working environment contaminated with dust, foreign bodies in the EAC, residence in tropical and humid climates and EAC cleansing with swabs, constitute the predisposing factors which collectively facilitate the germination of the spores and conidia of the most prevalent fungi [3, 4].

The most common genotypes that cause otomycosis are Candida and Aspergillus species [5]. Based on the infected strain, the fungi can manifest broad-spectrum pathogenesis and induce opportunistic infections by infiltrating the regular microbiota of the host body regions [6]. *Candida Tropicalis* and *Candida Albicans* are common infecting yeasts that result in various human diseases [7].

Clotrimazole or miconazole are the medications that are prescribed in conjunction with antibacterial drugs, such as ceftazidime, depending on the type of fungus [8]. However, certain compounds with disinfectant properties, such as boric acid and betadine, have also been employed along with miconazole [9]. When physicians’ recommendations are implemented in practice, complaints typically resolve within 1–2 weeks without adverse effects. In contrast, there are numerous reports available regarding the failure of otomycosis treatment, with a range of 5.88 to 17% [10, 11].

Silver has been increasingly used as a component of various medical devices with the intention of reducing infections. Silver nitrate was used topically throughout the 1800 s for the treatment of burns, ulcerations, and wound infections. It has been previously reported that silver is an essential component of conventional anti-fungal agents, exhibiting potent antimicrobial and inhibitory effects. The silver nitrate gel also has a good anti-oedematous action due to the hygroscopic property of hypromellose [12].

This investigation was designed to conduct a critical comparison of the efficacy of topical silver nitrate gel 0.2% and clotrimazole cream 1% in the treatment of otomycosis and to reduce the recurrence rate.

## Materials and methods

This prospective comparative study enrolled 200 adult patients aged 18–50 years with otomycosis attending the Outpatient Clinic of the Otorhinolaryngology Department at Benha University Hospitals from June 2024 to December 2024. Before the research, all participants provided written informed consent. The study was conducted in accordance with the principles of the Declaration of Helsinki. The study was conducted in accordance with the institutional ethical committee’s guidelines at Benha University Hospital (RC 6-5−2024).

Exclusion criteria were patients with chronic suppurative otitis media, with tympanic membrane perforation, uncontrolled diabetes mellitus (DM), systemic disease, respiratory tract infection, or radiation therapy, and those with a history of previous ear surgery.

### Grouping

Patients were allocated into two groups:

- Group (1) (*n* = 100): treated by applying silver nitrate gel 0.2% soaked Gelfoam.
- Group (2) (*n* = 100): treated by applying clotrimazole 1% cream-soaked gauze.

All patients underwent full history-taking, including age, sex, and comorbidities, and laboratory tests were additionally conducted. Patients who reported irritation, hearing loss, pain, sticky discharge, or a blocked sensation in the ear were examined using an otoscope. White, black, or brown fungal masses were visualized. After that, they were examined through aural microscopy. Sterile gauze was stretched over a Jobson Horne probe to purify the outer portion of the EAC. A sterile cotton swab was deeply inserted into the canal and gently rotated to capture fungal hyphae and discharge. A sterile culture tube was used to transport the sample to the microbiology section. The first swab was suspended in 1–2 drops of 10% potassium hydroxide with methylene blue (2:1) on a clean slide, and a cover glass was placed over it, avoiding air bubbles. It was examined under a microscope for the presence of fungal hyphae or yeast cells. The second swab was inoculated on two Sabouraud’s dextrose agar (SDA) plates with chloramphenicol. One plate was incubated at 22 °C and another at 37 °C for 1–2 weeks. Both plates were observed for fungal growth daily. Standard procedures identified fungal growth. Upon observation of growth on agar plates, direct microscopy was performed to determine fungal morphology using Lactophenol cotton blue stain. The third swab was inoculated on blood agar and MacConkey’s agar plates, incubated at 37 °C for 24–48 h, and examined for bacterial growth.

Following suction cleaning of the canal’s fungal mass, all potential detritus and discharge were simultaneously removed. Indicators of inflammation were observed through the myringitis and superficial epithelial exfoliation of the canal epidermis.

### In group (1)

The gel foam was cut and immersed in a 0.2% silver nitrate gel. Gelfoam sections were used to fill the canal completely, and the concha was packed with large sterile cotton. Patients were asked to return for a follow-up appointment every 5 days. The concha was stripped of its cotton on the 5th day. In the EAC, Gelfoam fragments were eliminated. The EAC and tympanic membrane were examined for any residual inflammation-related symptoms.

### In group (2)

The patients were provided with clotrimazole cream and instructed to leave it on for seven days. Subsequently, the EAC was cleaned of any remaining cream residue, and the otic conditions were reassessed.

In both groups, pain, edema and time to resolution were recorded. A 10-cm line was employed to assess pain using the visual analogue scale (VAS), which delineates a continuum between the two extremities; 0 represents the absence of pain on the left-hand side of the scale, while 10 represents the most severe pain on the right-hand side [13]. The incidence of complications, such as perforation, itching, and recurrence, was recorded. Post-application images were recorded on 1 day, 3days, and 1 week, separately for all patients. Each visit was followed up with an endoscopic examination. All patients were followed up for 2 months.

### Statistical analysis

Statistical analysis was done by SPSS v28 (IBM Inc., Armonk, NY, USA). For comparison between two groups, the unpaired Student’s t-test was used to compare quantitative parameters, which were presented as mean ± SD. Chi-square test or Fisher’s exact test, when appropriate, was used to compare qualitative variables, which were presented as frequency and percentage (%).Within‑group comparisons across different time points were conducted using Cochran’s Q test for categorical outcomes, with McNemar’s test applied for pairwise contrasts. A two-tailed P value < 0.05 was considered statistically significant.

## Results

Table 1 shows that the age, sex, and laterality of infection were comparable between the studied groups.

**Table 1:** Baseline characteristics of the studied groups

		Group (1) (*n* = 100)	Group (2) (*n* = 100)	P value
Age (years)		34.23 ± 8.79	34.63 ± 9.13	0.753
Sex	Male	66 (66%)	58 (58%)	0.243
	Female	34 (34%)	42 (42%)	
Laterality of infection	Right	47 (47%)	52 (52%)	0.479
	Left	53 (53%)	48 (48%)	

Data presented as mean ± SD or frequency (%), *: statistically significant as p value < 0.05

Regarding the causative pathogen, Aspergillus. Flavus was significantly the most common pathogen in group (1) compared to group (2) (*P* < 0.001), while *Candida Albicans* was significantly the most common pathogen in group (2) compared to group (1) (*P* = 0.002), with no significant difference between both groups regarding the other causative pathogens. Table 2.

**Table 2:** Causative pathogen of the studied groups

		Group (1) (*n* = 100)	Group (2) (*n* = 100)	P value
Pathogen	*Aspergillus. Niger*	14 (14%)	22 (22%)	0.141
	*Aspergillus. Flavus*	43 (43%)	18 (18%)	< 0.001*
	*Aspergillus. Fumigatus*	20 (20%)	15 (15%)	0.352
	*Candida. Albicans*	16 (16%)	35 (35%)	0.002*
	*Candida. Tropicalis*	4 (4%)	6 (6%)	0.747
	*Candida. Krusei*	14 (14%)	22 (22%)	0.141

Data presented as frequency (%), chi-square test was used, and Fisher’s exact test was also used when applicable, *: statistically significant as p value < 0.05

In group (1), the edema had been significantly improved during the follow-up visits (33%, 2%, 0% respectively, *P* < 0.001). Pain had been significantly decreased during the follow-up visits (25%, 3%, 0% respectively, *P* < 0.001). Itching had been significantly improved during the follow-up visits (32%, 5%, 0% respectively, *P* < 0.001). In group (2), the edema had been significantly improved during the follow-up visits (35%, 11%, 0% respectively, *P* < 0.001). Pain had been significantly decreased during the follow-up visits (36%, 11%, and 4%, respectively; *P* < 0.001). Itching had been significantly improved during the follow-up visits (34%, 1%, 0% respectively, *P* < 0.001). Table 3.

**Table 3:** Clinical symptoms of the studied groups

		Group (1) (*n* = 100)	Group (2) (*n* = 100)	P value
Edema	1 day	33 (33%)	35 (35%)	0.881
	3 days	2 (2%)	11 (11%)	0.018*
	1 week	0 (0%)	0 (0%)	----
	P-value within group	< 0.001*	< 0.001*	
Pain	1 day	25 (25%)	36 (36%)	0.091
	3 days	3 (3%)	11 (11%)	0.026*
	1 week	0 (0%)	4 (4%)	0.043*
	P-value within group	< 0.001*	< 0.001*	
Itching	1 day	32 (32%)	34 (34%)	0.763
	3 days	5 (5%)	1 (1%)	0.118
	1 week	0 (0%)	0 (0%)	----
	P-value within group	< 0.001*	< 0.001*	

Data presented as frequency (%), chi-square test was used, and Fisher’s exact test was also used when applicable for the comparison of both groups, while chi was used for multiple comparisons, *: statistically significant as p value < 0.05

Table 3 shows that group (1) had significantly lower edema at 3 days than group (2) (*P* = 0.018), with no significant difference between the groups at 1 day, and both groups had no edema at 1 week. In group (1), pain at 3 days and 1 week was significantly lower than in group (2) (*P* = 0.026, 0.043), with no significant difference between the two groups at 1 day. Itching at 1 and 3 days was insignificantly different between the two groups.

The mean time of resolving was 3 ± 0.82 days in group (1) and was 7.05 ± 0.82 days in group (2). Regarding the complications, perforation occurred in only 1 (1%) patient in group (1) and none in group (2). In addition, recurrence was observed in 2 (2%) patients in group (1) and 15 (15%) patients in group 2. Group (1) resolved significantly faster than group (2) (*P* < 0.001). Recurrence rates were significantly lower in group (1) than in group (2) (*P* < 0.001). There was no significant difference between the two groups according to perforation. Table 4.

**Table 4:** Outcomes of the studied groups

		Group (1) (*n* = 100)	Group (2) (*n* = 100)	P value
Time of resolving (days)		3.00 ± 0.82	7.05 ± 0.82	< 0.001*
Complications	Perforation	1 (1%)	0 (0%)	1.00
	Recurrence	2 (2%)	15 (15%)	< 0.001*

Data presented as mean ± SD or frequency (%), *: statistically significant as p value < 0.05

## Discussion

Otomycosis primarily affects the squamous epithelium of the external ear canal, leading to injury and symptoms such as itching, aural fullness, and discomfort. The most common symptoms are irritation and fullness, which are similar to those found in the current study. EAC is oversensitive and enlarged [14, 15].

Regarding the causative pathogen, Aspergillus. Flavus was significantly the most common pathogen in group (1) compared to group (2) (*P* < 0.001), while *Candida Albicans* was significantly the most common pathogen in group (2) compared to group (1) (*P* = 0.002), with no significant difference between both groups regarding the other causative pathogens.

Aspergillus and Candida were the most prevalent infectious fungal isolates. Various studies have reported a higher prevalence of Aspergillus (Niger, flavus, and other species) as agents [16, 17].

A parallel finding was stated by Keyvan et al. [18], where the most prevalent infectious agent was A. flavus (63.33%), Candida was responsible for otomycosis in only 12.66% of the total cases. Conversely, Jaiswal et al. [19] Identified 46% of Candida species.

Predisposing factors are not addressed as initial treatment for otomycosis. Following this, it is advised to administer an antifungal agent (local or systemic) and perform local cleansing [20, 21]. According to some studies, the infection can be effectively treated by administering ear drops 3 to 4 times daily for 5 to 7 days to reduce the acidity of the ear canal. Conversely, other researchers recommend that the ear be filled with an antifungal agent daily [22, 23].

Vennewald and Klemm [24] determined that topical antifungals, including cyclopiroxolamine, clotrimazole, miconazole, tolnaftate, and bifonazole, can be administered effectively to treat non-invasive fungal otitis externa following local cleansing. Alternatively, fungal malignant otitis externa can be exacerbated by meningitis and mastoiditis. This condition should be managed with oral antifungal agents, including voriconazole, itraconazole, and posaconazole, as they can cross the blood-brain barrier and enter bone cells.

Based on its broad-spectrum antimycotic activity, Clotrimazole can be classified as an imidazole derivative as it disrupts the biosynthesis of ergosterol, which is a key constituent of the cell membranes of yeast and fungi. The fungal cytochrome P450 enzyme lanosterol-14-demethylase uses it as a substrate to catalyse the conversion of lanosterol to ergosterol, thereby replacing lanosterol as a precursor. As a result, the permeability of fungal cell walls is modified. In addition to its antimycotic properties, Clotrimazole preparations are believed to be safe, with no evidence of ototoxicity [25].

Mofatteh et al. [26] investigated 204 patients diagnosed with otomycosis. They compared the recovery rates of topical betadine (povidone-iodine) and clotrimazole and found that both had the same effect. Romsaithong et al. [27] randomly divided 120 otomycosis patients into 2 groups, patients received 1% clotrimazole solution (intervention group), or patients received 3% boric acid in 70% alcohol (control group). They revealed that the treatment of otomycosis is more effective with a 1% clotrimazole solution than with a 3% boric acid solution in 70% alcohol. Dundar et al., [28] locally applied 1% clotrimazole cream to the external auditory canal during a single appointment. No compliance or exhaustive visits for local purification are required from the patient, so it is more convenient than prescribing topical drops.

A silver nitrate gel was chosen in order to provide a sustained release of silver ions to the ear canal skin as the gel dries up, and also to prevent the silver nitrate from running out of the ear, which would cause temporary dark stains on the skin and permanent stains on textiles. The beneficial effects of silver nitrate in reducing or preventing infection have been observed in the topical treatment of ophthalmia neonatorum [29].

Gel production is effortless and inexpensive. A single 1 mL instillation contains ingredients priced at a few eurocents. Most of the time, a solitary instillation is adequate. The hygroscopic property of Hypromellose also contributes to the silver nitrate gel’s effective anti-oedematous effect [30].

Hasselt et al. [30] employed a single application of silver nitrate gel for otitis externa treatment. It was observed that this type of treatment saved both time and money. Consequently, Dundar et al. [28] believed that in a single visit, the external auditory meatus is filled with 1% clotrimazole after local purification, which is a cost-effective and effective treatment method. Patient compliance is believed to be inadequate due to the need to administer antifungal medications three to four times daily. Additionally, the high patient compliance makes this treatment superior to long-term topical therapy.

Group (1) exhibited a significantly reduced recurrence rate than group (2) (*P* < 0.001), with 2 (2%) patients in group (1) and 15 (15%) patients in group (2) that experienced recurrence.

Several studies have suggested that otomycosis may relapse. Only one study has reported no relapses [31]. Otomycosis relapse was 48% in reported study [32], and 14.29% in another [33]. Otomycosis relapse has been reported in the majority of studies to be less than 9% (7.1–8.8%), except in a few investigations [25, 34]. Overall, the comparison of these results indicates that the use of silver nitrate gel and clotrimazole drop leads to a decrease in the rate of relapse of otomycosis in Kiakojuri et al., [25] Study.

It is rare to find studies that compare the efficacy of silver nitrate and clotrimazole in the treatment of otomycosis.

However, this study exhibited certain limitations, including being a single-center study and a lack of randomization, which increases the risk of selection bias and confounding, thereby limiting the internal validity of the study and particularly given the significant difference in pathogen distribution between groups. One of the major limitations that our findings should be interpreted with caution, as fungal diversity may influence treatment outcomes and recurrence rates, therefore, observed superiority of silver nitrate may be confounded by pathogen distribution. Additionally, limited information is available regarding the antifungal properties of the silver gel commonly used to treat otomycosis. Short follow-up duration for recurrence assessment. Absence of audiometric evaluation to confirm ototoxic safety, limited generalizability due to exclusion of pediatric and immunocompromised populations.

## Conclusions

Both silver nitrate gel 0.2% and clotrimazole 1% cream were effective in the treatment of otomycosis. Silver nitrate gel was superior to clotrimazole, with earlier resolution and fewer/minimal recurrences.

Additional research with larger samples is necessary to verify the advantages of silver nitrate gel.
